# Neural correlates of prospective memory in college students with anxiety

**DOI:** 10.3389/fpsyg.2024.1430373

**Published:** 2024-09-24

**Authors:** Michaela Rice, Melissa Hansen, Michael L. Thomas, Deana Davalos

**Affiliations:** Cognitive Neuroscience Program, Department of Psychology, Colorado State University, Fort Collins, CO, United States

**Keywords:** prospective memory (PM), event-related potentials, cue detection, intention retrieval, electroencephalography

## Abstract

**Introduction:**

Prospective memory (PM) is the ability to create and execute future tasks. It is comprised of two components: cue detection and intention retrieval. PM is essential for performing high-level goals, a proficiency extremely important in college populations. Anxiety is a prevalent psychological experience in college populations that may be associated with impairments in PM. The present study examined PM performance and anxiety in college students, using neurophysiology to measure the mechanism of impairment.

**Methods:**

After self-reporting anxiety levels, 80 participants completed an event-based, focal PM task while two event-related potentials were recorded from an electroencephalogram: the N300 to assess cue detection, and the prospective positivity to assess intention retrieval.

**Results:**

The results demonstrated that, when controlling for age and gender, higher state anxiety was significantly associated with lower PM accuracy (*β* = −0.27, *p* = 0.020) and lower prospective positivity amplitude (*β* = −0.04, *p* = 0.021). Lower prospective positivity amplitude was significantly associated with lower PM accuracy (*β* = 0.27, *p* = 0.015). Higher state anxiety was significantly indirectly associated with lower PM accuracy mediated by lower prospective positivity amplitude (ab = −0.11, *p* = 0.047).

**Discussion:**

These findings suggest intention retrieval could be a key component in supporting PM for college students with high state anxiety.

## 1 Introduction

Prospective memory (PM) is the ability to create and perform future-intended tasks and goals (Einstein and McDaniel, 1990). Real-world applications of PM include remembering to take medication at specific times throughout the day, remembering to buy groceries after work, and remembering to submit assignments for school on time (Einstein and McDaniel, 2005). PM plays an important role in autonomy and independence, and impairments can have serious ramifications for a person’s health, independence, and quality of life (Guynn et al., 1998; Kinsella et al., 2009; Ordemann et al., 2014; Ramos et al., 2020). It is critical for researchers to understand which populations are at risk for PM deficits, as this knowledge can help people receive the necessary intervention and reinforcement for their PM abilities, health, and overall well-being.

Prospective memory can be bisected into two components: a prospective component and a retrospective component (Einstein and McDaniel, 1990; McDaniel and Einstein, 1992). The prospective component (i.e., cue detection) refers to a person noticing the appropriate cue for carrying out their intended goal. The retrospective component (i.e., intention retrieval) refers to a person retrieving the intended goal from their memory (Brandimonte et al., 2014; McDaniel and Einstein, 1992; Smith and Bayen, 2004). If the PM goal is to take medication when eating a meal, cue detection would be the person eating the meal and realizing they must carry out their goal; intention retrieval is the act of remembering to then take their medication. PM goals can be event-based or time-based, depending on the type of cue is involved in the task. An event-based task is cued when a particular event occurs, while a time-based task is cued by a specific time of day (Cona et al., 2012). The previous example indicates an event-based PM goal, as will be the focus of the current study.

Event-related potentials (ERPs) are particularly informative when studying PM, as the high temporal resolution is useful in parsing the two components of PM. Cue detection and intention retrieval can be characterized through the N300 and the prospective positivity (Hering et al., 2016; West and Krompinger, 2005). The N300 likely represents cue detection: it is denoted by phasic negativity over the occipital–parietal region between 200 and 400 ms after stimulus presentation (Hering et al., 2016; West et al., 2001; West et al., 2003; West and Ross-Munroe, 2002). The prospective positivity likely represents intention retrieval: it is distinguished by sustained positivity over the parietal region between 500 and 1,000 ms after stimulus presentation (Cona et al., 2012; Hering et al., 2016; West et al., 2001; West and Ross-Munroe, 2002). Previous work has highlighted the importance of other ERP components and frequencies including the P3b for stimulus evaluation, the FN400 for stimulus recognition, the frontal slow wave for retrieval monitoring processes, and alpha and beta bands, demonstrating the complex neurocognitive underpinnings of PM (Cona et al., 2012; Cona et al., 2014; Laera et al., 2021). By using ERPs, researchers can examine if the components of PM help explain or predict overall PM impairments in various populations.

Individuals with high levels of anxiety are one population that researchers have begun to examine regarding PM abilities. Researchers are interested in anxiety because of its well-known harm on people’s emotional, cognitive, and physical well-being as a commonly diagnosed psychological disorder (Andrews and Hicks, 2017; Aquin et al., 2017; Robinson et al., 2013; Shah and Pol, 2020). Anxiety is especially prevalent among undergraduate college students. According to the American College Health Association (2018, 2022), 63% of undergraduate students have reported experiencing overwhelming levels of anxiety, and 35% of undergraduates have been diagnosed with an anxiety disorder. College students require a high level of cognitive functioning, including PM abilities, to succeed academically and psychologically. Understanding how anxiety may interact with forms of cognition, such as PM could help us support this population in critical ways. While a handful of studies have reported a potentially positive or neutral relationship between PM and anxiety levels (Nigro and Cicogna, 1999; Kliegel and Jäger, 2006; Notebaert et al., 2019), most studies demonstrate an inverse association between PM and anxiety for undergraduate students (Bowman et al., 2019; Arnold et al., 2015; Harris, 1999; Harris and Cumming, 2003; Kliegel and Jäger, 2006; Cuttler and Graf, 2009).

Almost all research has examined the role of anxiety in PM without separating cue detection and intention retrieval, but Arnold et al. (2015) used multinomial processing tree modeling to investigate how state and trait anxiety relate to the two components of PM. They observed a negative correlation between state anxiety and PM accuracy, as well as a negative relationship between state anxiety and cue detection (Arnold et al., 2015). These results suggested that cue detection may be a particularly sensitive component of PM for college students with high levels of state anxiety. To date, there are no studies that have implemented neurophysiology to examine similar aims. Researchers can measure ERPs during a PM task to investigate the neural processes underlying the two components of PM (Chen et al., 2015). By utilizing ERPs, the current study extends upon previous behavioral findings by examining neural correlates associated with the two components of PM in college students with anxiety.

The purpose of this study was to examine the relationship between PM and anxiety in college students using ERPs. Undergraduate students with varying levels of anxiety performed a PM ERP task to assess overall PM performance and investigate whether anxiety symptoms interacted differently with the two components of PM (cue detection and intention retrieval) as measured by the N300 and the prospective positivity. This methodology allowed researchers to parse the neural mechanism behind PM deficits in college students with anxiety.

Based on previous research indicating that PM abilities are impaired in college students with high anxiety levels (Arnold et al., 2015; Bowman et al., 2019), we hypothesized that anxiety levels would negatively predict PM accuracy. Further, we hypothesized that N300 and the prospective positivity would be predicted by anxiety. Based on Arnold et al. (2015), we tentatively expected to see that cue detection, assessed by the N300, would likely be especially vulnerable to anxiety levels. More specifically, anxiety levels would negatively predict the N300 amplitude, and the N300 amplitude would positively predict PM accuracy. We explored the role of the prospective positivity in PM performance and anxiety also, to thoroughly characterize the neural correlates of PM in college students with anxiety.

## 2 Methods

### 2.1 Participants

Data for this study were collected from Department of Psychology undergraduate students at Colorado State University in 2022. Prior to coming to the lab, potential participants completed a demographics survey assessing sex assigned at birth, gender, age, race, ethnicity, and history of epilepsy. Exclusion criteria included not having normal or corrected hearing and vision and a history of seizures. To establish variability in anxiety levels in the sample, potential participants were screened into the study based on the trait anxiety scores via the trait subscale of the State–Trait Anxiety Inventory (STAI-T; Spielberger et al., 1983). The STAI-T was used as a screener to assess longstanding, characteristic anxiety (Julian, 2011; Notebaert et al., 2019; Xia et al., 2020). Individuals scoring in the upper and lower quartile of the distribution of anxiety scores were included in the study (Xia et al., 2020).

Ninety-nine students were selected based on their STAI-T scores. Eight participants were excluded due to not understanding the task, with an accuracy lower than 10% on the PM trials. Eleven participants were excluded due to no longer fitting in the upper or lower anxiety quartiles, since the cutoff scores shifted as more potential participants completed the screener. With these exclusions, there was final *n* of 80 (*M*_age_ = 20.12, SD_age_ = 3.11, 9 left-handed, gender identity: 56 women, 5 gender fluid individuals, 1 transgender woman, 1 transgender man). See Table 1 for the full demographics of the sample.

**Table 1:** Descriptive statistics.

Variable name	*n*	Percent	Mean	Standard deviation	Standard error	Min.	Max.	Range
Age	–	–	20.12	3.11	0.35	18	35	17
Gender identity								
Man	17	21.25						
Woman	56	70.00						
Transgender man	1	1.25						
Transgender woman	1	1.25						
Gender fluid	5	6.25						
Race								
White	71	88.75						
Black or African American	3	3.75	–	–	–	–	–	–
Asian	5	6.25						
Native American or Alaska Native	1	1.25						
Ethnicity								
Not Hispanic of Latinx	73	91.25						
Hispanic or Latinx	7	8.75						
BAI Score	–	–	19.98	13.73	1.54	0	56	56
STAI-S Score	–	–	39.39	11.44	1.28	20	63	43
STAI-T Score	–	–	45.92	15.31	1.68	20	74	54
PM Accuracy	–	–	70.73	23.24	2.40	11.11	100	88.89

### 2.2 General procedure

The study was reviewed and approved by Colorado State University’s Social, Behavioral, and Educational Research Institutional Review Board (IRB #2590, approved November 21st, 2021).

Participants provided informed consent and then completed the state (STAI-S) and trait subscales of the STAI (Spielberger et al., 1983) and the Beck Anxiety Inventory (BAI; Beck and Steer, 1993). Next, participants were fitted with the EEG cap and provided with task instructions to complete the focal event-based PM task. Following completion of the task, participants were debriefed and provided with course credit/extra credit.

### 2.3 Measures

State–Trait Anxiety Inventory (Spielberger et al., 1983): participants reported their state anxiety by indicating “Not at all” to “Very much so” to a series of 20 statements (e.g., “I feel nervous”). Participants also reported their trait anxiety by indicating “Not at all” to “Very much so” to another series of 20 statements (e.g., “I am content”). The STAI has high internal reliability (*α* = 0.86–0.95), test–retest reliability (*α* = 0.65–0.75), and concurrent validity (Spielberger et al., 1983). A higher score on the STAI was indicative of higher anxiety.

Beck Anxiety Inventory (BAI; Beck and Steer, 1993): participants reported their general anxiety levels by indicating “0 = Not at all,” “1 = Mildly but it did not bother me much,” “2 = Moderately – it wasn’t pleasant at times,” “3 = Severely it bothered me a lot” to a series of 22 statements (e.g., “Wobbliness in legs,” “Fear of worst happening,” “Fear of losing control”). The BAI has high internal reliability (*α* = 0.92), and convergent and discriminant validity (Beck et al., 1988; McDaniel and Einstein, 1992). Higher BAI scores represented higher anxiety in college students.

Visual Search PM Task: this task was created using PsychoPy3 Experiment Builder (v2021.2.3; Peirce et al., 2019). The task was adapted from West and Wymbs (2004) and has three essential components: an ongoing visual search task, a cue detection component, and an intention retrieval component. During the ongoing visual search task, participants were shown a target letter in the center of the computer screen, followed by two test letters on the left and right sides of the display. If the target letter was present in the test letters, the participant pressed V with their left index finger. If the target letter was not present in the test letter, the participants pressed B with their right index finger. The PM components were introduced to the visual search task after the participants completed a block of the isolated visual search task. The letters D and M serve as the cue detection components of PM task. If the letters D or M were present in the test letters, the participants were instructed to select N with their right middle finger. Remembering to select N when prompted with D or M was the intention retrieval component of the PM task. This PM paradigm has been validated in previous research to elicit the desired ERP components (West and Wymbs, 2004).

The task began with instructions for the visual search task, without the PM components: there was 1 practice block of 10 trials and 1 test block of 96 trials. The test block without PM included 24 target-present trials shown on the left side, 24 target-present trials shown on the right side, and 48 target absent trials.

The instructions for incorporating the PM components were shown and explained by the researcher after 1 block of the visual search task. This procedure ensured the participants understood the ongoing visual search component and could appropriately integrate the PM components. The task included 1 practice block of 10 trials, and 3 test blocks of 168 trials each. The test block included 36 target-present trials shown on the left side, 36 target-present trials shown on the right side, and 72 target absent trials, 12 prospective-cue trials shown on the left side, and 12 prospective cue-trials on the right side. The prospective-cue and target were never presented together in a trial. The task ended with another test block of 96 trials for the visual search task without the PM components.

Each trial included a target, a fixation, and a rest period. For the target display, the target letter was presented in the center of the computer monitor for 150 ms. The target was replaced by a fixation cross for 850 ms, followed by the test display. The test display included two letters presented on the left and right sides, with a fixation cross in the middle. The letters were presented in white, Arial font, 1/10 the size of the screen. They were separated by 35 mm of gray space. The test display was presented for 400 ms and then replaced by a blank gray screen until the response was made. If the participants did not respond after 5,000 ms, the task continued. The screen remained blank for 500 ms after the response, and then the target letter for the next trial was presented. For the first trial of each block, the screen was blank for 1,000 ms after the space bar was pressed to initiate the block (West and Wymbs, 2004). See Rice (2023) for a more detailed description of the study materials and protocol.

### 2.4 EEG procedure

The EEG was recorded at a rate of 1,000 Hz using SynAmps2/RT 64 Channel system (Curry7, Compumedics NeuroScan, United States) (acquisition bandwidth = 1,000 Hz, sampling rate = 1,000 Hz) and a 64 electrode Quik-cap Neo Net with sewn-in or affixed to skin via an adhesive patch silver-chloride sintered electrodes (Diameter = 1.5 cm; Compumedics USA, Inc. Model: C190). The electrodes are placed in the cap based on the extended 10/20 electrode placement standard. A 0.01 Hz high-pass filter and a 100 Hz low-pass filter were used during recordings. The impedance was kept below 20 kΩ. Electrode placements followed the Quik-cap 64 system and included all electrodes (Fpz, Fz, Pz, Poz, Oz, Fp1, Fp2, Af3, Af4, F1, F2 F3, F4, F5, F6 F7, F8, F11, F12, Fc1, Fcz, Fc2, Fc3, Fc4 Fc5, Fc6, Ft11, Fc12, C1, Cz, C2, C3, C4, C5, C6, T7, T8, Tp7, Tp8, Cp1, CPz, Cp2, Cp3, Cp4, Cp5, Cp6, P1, P2, P3, P4, P5, P7, P6, P8, Po3, Po4, O1, O2, Po7, Po8, Cb1, Cb2, left mastoid M1, right mastoid M2, VEOU, VEOL, HEOL, HEOR). Eye movement was monitored with electrode placement on the lateral and superior of the left eye for vertical electrooculogram (EOG), and the outer corner of each eye for horizontal EOG. The reference electrode was placed between Cz and CPz. The ground electrode was AFz. During recording, all electrodes were referenced to the electrode between Cz and CPz (Rice, 2023).

#### 2.4.1 EEG/ERP analyses

N300: cue detection was quantified as a single continuous variable calculated from the average amplitude (μV) of the N300 for correct prospective-cue trials between 200 and 400 ms, from electrodes O1, Oz and O2. A stronger negative amplitude indicated an enhanced N300.

Prospective positivity: intention retrieval was quantified as a single continuous variable calculated from the average amplitude (μV) of the prospective positivity for correct prospective-cue trials between 500 and 1,000 ms, from electrodes P3, Pz and P4. A stronger positive amplitude indicated an enhanced prospective positivity.

The EEG data were processed in MATLAB (RRID:SCR_001622) using EEGLAB (RRID:SCR_007292) with the ERPLAB (RRID:SCR_009574) plug-in. A 0.1–30 Hz zero-phase-shift bandpass filter was applied. Ocular artifacts were corrected using the Independent Component Analysis feature in EEGLAB. The EEG data were re-referenced to an average reference and trials exceeding a voltage of ±100 μV were removed from trials. The ERP epoch included −200 to 1,200 ms of activity around stimulus onset (Rice, 2023).

To analyze the electrode clusters as opposed to individual electrode responses, mean voltages of electrodes O1, Oz, and O2 were averaged to measure the N300. Mean voltages of electrodes P3, Pz, and P4 were averaged to measure the prospective positivity, as has been performed in previous literature (Chen et al., 2015; Groot and Strien, 2019; Zhang et al., 2019).

### 2.5 Statistical methods

Multiple linear regression analyses were conducted in R (version 4.1.1) to examine associations among state and trait anxiety, the N300 amplitude, the prospective positivity amplitude, and prospective memory accuracy with participant age and gender as control variables in the models. Gender and age were controlled for due to anxiety levels, PM performance, and ERPs varying with age and gender (Lenze and Wetherell, 2011; Koo et al., 2021; McLean et al., 2011; Riess et al., 2016; Bowman et al., 2015; Guillem and Mograss, 2005). Research suggests that ERP components associated with cognitive functioning can differ across age groups, particularly between adolescence and adulthood (Davies et al., 2004; Ladouceur et al., 2007; Manzi et al., 2011). Age-related variations in neural processing and cognitive functions may impact ERP measures, potentially confounding the relationships under investigation. To address this, we controlled for age in our analyses to more precisely isolate the effects of anxiety on ERPs and prospective memory performance, ensuring that our findings were not skewed by age-related variability. Self-reported gender was used as opposed to sex, due to previous studies reporting gender differences in anxiety levels among cisgender men, cisgender women, and transgender individuals (McLean et al., 2011; Reisner et al., 2016). Gender was separated into three categories to account for individuals outside the gender binary (e.g., man, women, and transgender + non-binary) and dummy coded in regression analyses (DuBois and Shattuck-Heidorn, 2021). All tests were two tailed (*α* = 0.05). Effect sizes were calculated from the linear regressions, measured by a partial R-squared. First, the R-squared values were obtained for both the full models and reduced models (e.g., without the independent variable). Then, the partial R-squared was calculated as the difference between these R-squared values, providing a measure of the unique effect size of the independent variables while accounting for the control variables of age and gender.

Based on the results of the multiple linear regression, exploratory mediation analyses were then conducted to examine the extent to which the significant ERP might explain associations between anxiety and prospective memory. Mediation analyses were performed using the “mediation” package in R (Tingley et al., 2014). For the significant ERP findings, the following analyses were conducted. First, two regression models were fitted: the mediator model for the conditional distribution of the mediator (e.g., prospective positivity) given the independent variable (e.g., state anxiety), and the outcome model for the conditional distribution of the outcome (e.g., prospective memory) given the independent variable and the mediator. Subsequently, the outputs of these two regression models served as the main inputs to the “mediate” function that computes the direct, indirect, and total effects. The significance of the indirect effect was estimated by the non-parametric bootstrap approach (with 10,000 random samplings). The analyses also controlled for the covariates in the regression models, age and gender, ensuring that the mediation effects observed were not confounded by these variables. All assumptions of the mediation model, including linearity, were checked, and no violations were detected, lending confidence to the interpretation of the mediation effects.

## 3 Results

### 3.1 Descriptive statistics

Descriptive statistics for anxiety measures, prospective memory, the N300, and the prospective positivity, and zero-order correlations between these variables are provided in Table 2. Notably, gender was correlated with all three anxiety measures (all *p* < 0.01). Age was not correlated with any of the variables of interest.

**Table 2:** Means, standard deviations, and correlations with confidence intervals.

Variable	*N*	*M*	*SD*	1	2	3	4	5	6
1. STAIS	80	39.39	11.44						
2. STAIT	80	45.92	15.06	0.80** [0.71, 0.87]					
3. BAI	80	19.89	13.73	0.71** [0.59, 0.81]	0.76** [0.65, 0.84]				
4. PP	80	2.09	1.75	−0.27* [−0.46, −0.05]	−0.17 [−0.38, 0.05]	−0.16 [−0.37, 0.06]			
5. N300	80	0.64	3.44	−0.07 [−0.29, 0.15]	−0.08 [−0.30, 0.14]	−0.10 [−0.31, 0.12]	0.36** [0.15, 0.54]		
6. Accuracy	80	70.73	21.49	−0.21 [−0.41, 0.02]	−0.10 [−0.32, 0.12]	−0.02 [−0.24, 0.20]	0.23* [0.01, 0.43]	0.17 [−0.05, 0.38]	
7. Age	80	20.12	3.11	−0.06 [−0.27, 0.17]	−0.11 [−0.32, 0.11]	−0.06 [−0.27, 0.16]	−0.12 [−0.33, 0.10]	0.11 [−0.11, 0.32]	0.14 [−0.08, 0.35]
8. Gender^1^	73	–	–	0.16 [−0.07, 0.37]	0.31** [0.08, 0.50]	0.27* [0.05, 0.47]	−0.05 [−0.28, 0.18]	−0.12 [−0.34, 0.11]	−0.03 [−0.26, 0.20]
9. Gender^2^	63	–	–	0.33** [0.09, 0.54]	0.34** [0.10, 0.54]	0.34** [0.10, 0.54]	−0.12 [−0.35, 0.14]	−0.16 [−0.39, 0.09]	0.30* [0.06, 0.51]
10. Gender^3^	27	–	–	0.63** [0.31, 0.82]	0.78** [0.55, 0.90]	0.74** [0.49, 0.88]	−0.25 [−0.59, 0.17]	−0.32 [−0.64, 0.09]	0.38 [−0.03, 0.68]

*M* and SD are used to represent mean and standard deviation, respectively. Point-biserial correlation coefficients were calculated to assess the relationships between the binary categorical variable (gender) and the continuous variables. Pearson’s correlation coefficients were calculated to evaluate the correlations between the continuous variables. Values in square brackets indicate the 95% confidence interval for each correlation. The confidence interval is a plausible range of population correlations that could have caused the sample correlation (Cumming, 2014). * indicates *p* < 0.05, ** indicates *p* < 0.01. Gender^1^: Man = 0, Woman = 1. Gender^2^: Women = 0, Transgender + non-binary = 1. Gender^3^: Men = 0, Transgender + non-binary = 1.

The normal range of the STAI-S and STAI-T is 20–80. The range for the STAI-S and STAI-T for moderate anxiety is 38–44 and high anxiety is 45–80. In this sample, the average STAI-S score fell into the category of moderate anxiety scores of state anxiety (see Table 1). Forty six percent of participants fell into the low anxiety category, 19% into moderate anxiety, and 35% into the high anxiety category. The average STAI-T score fell into the category of high anxiety scores of trait anxiety (see Table 1). Forty percent were classified as low anxiety, 9% were classified into moderate anxiety and 51% were classified as high anxiety.

The normal range of the BAI is 0–63. The range for the BAI for moderate anxiety is 16–25 and high anxiety is 26–63. In this sample, the average BAI score fell into the category of moderate anxiety scores of state anxiety (see Table 1). Forty four percent were classified as low anxiety, 10% were classified into moderate anxiety and 36% were classified as high anxiety.

### 3.2 Main effects of anxiety and prospective memory

**Figure 1:**
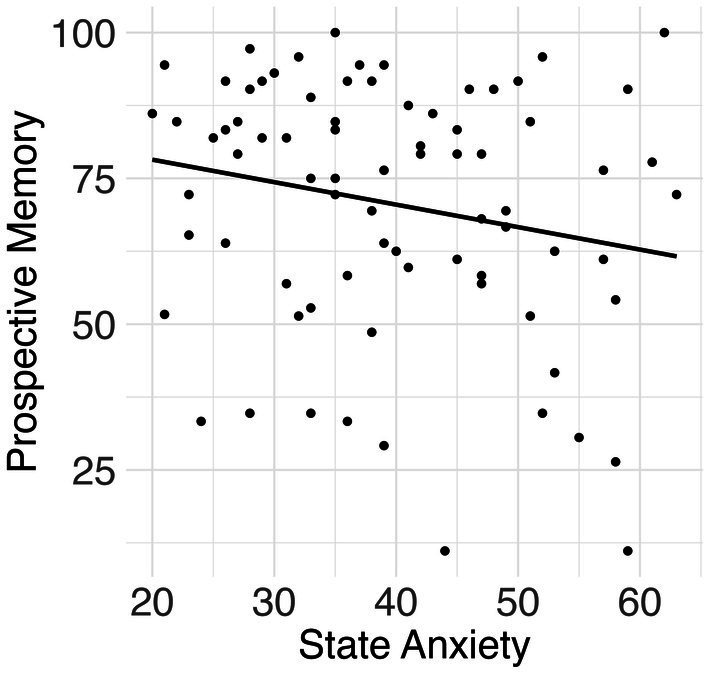
Results for State Anxiety and Prospective Memory Accuracy. A scatter plot showing an increase in state anxiety associated with a decrease in prospective memory.

The results revealed a significant inverse association between state anxiety and PM (*β* = −0.27, *p* = 0.020, *η*^2^ = 0.090) (see Figure 1). Trait anxiety and BAI were not significantly associated with PM (*β* = −0.19, *p* = 0.131, *η*^2^ = 0.029; *β* = −0.09, *p* = 0.463, *η*^2^ = 0.007). All analyses controlled for age and gender.

### 3.3 Main effects of anxiety and ERPs

**Figure 2:**
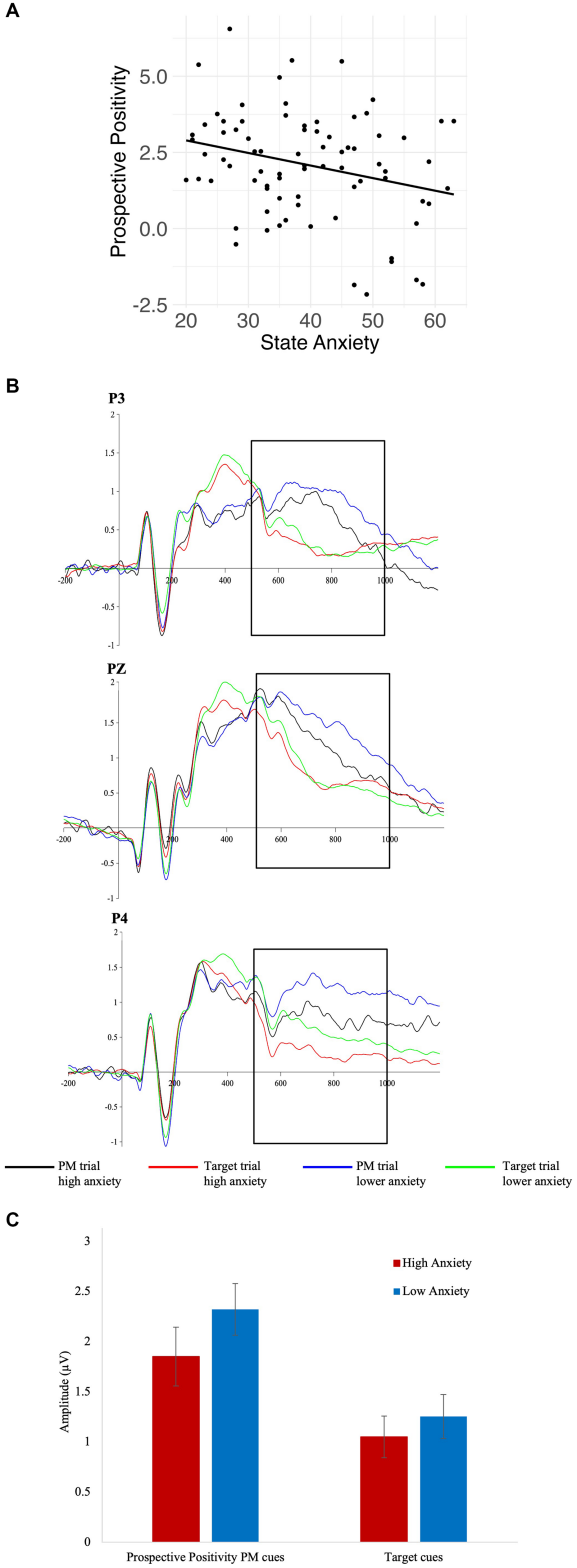
Results for State Anxiety and Prospective Positivity Amplitude. **(A)** A scatter plot showing an increase in state anxiety associated with a decrease in prospective positivity. **(B)** ERP results for the prospective positivity at electrodes P3, Pz, and P4. The prospective positivity is characterized by a sustained positive peak 500 – 1000 ms after stimulus onset. At all electrodes, the neural response during the PM trials for high anxiety participants is diminished in comparison to the low anxiety participants. This decreased amplitude suggests potential struggles with the intention retrieval component of PM for high anxiety participants. **(C)** A bar plot demonstrating the average amplitudes and standard error of the mean (SEM) for the prospective positivity PM trials and the target trials. While group comparisons between low and high anxiety were not calculated, **(B)** and **(C)** provide visualizations of the associations and inter-participant variability.

There was a significant inverse association between state anxiety and the prospective positivity (*β* = −0.27, *p* = 0.021, *η*^2^ = 0.058), while controlling for age and gender (see Figure 2). Trait anxiety and BAI were not significantly associated with the prospective positivity (*β* = −0.17, *p* = 0.168, *η*^2^ = 0.024; *β* = −0.15, *p* = 0.240, *η*^2^ = 0.018) or the N300 (*β* = 0.02, *p* = 0.879, *η*^2^ = 0.000; *β* = −0.02, *p* = 0.875, *η*^2^ = 0.000). State anxiety was not significantly associated with the N300 (*β* = −0.00, *p* = 0.968, *η*^2^ = 0.000). All analyses controlled for age and gender.

### 3.4 Main effects of ERPs and prospective memory

**Figure 3:**
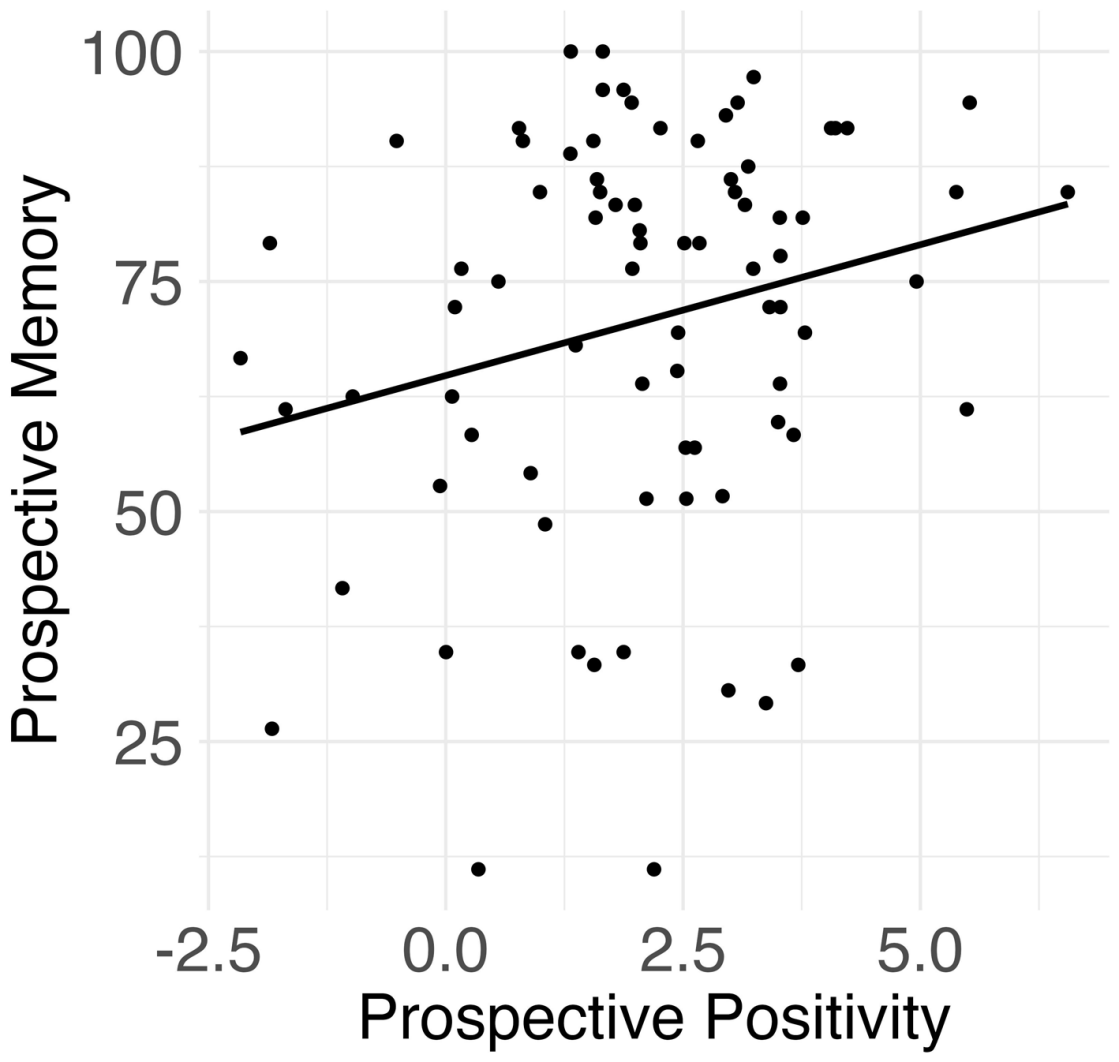
Results for Prospective Positivity Amplitude and Prospective Memory Accuracy. A scatter plot showing an increase in prospective positivity associated with an increase in prospective memory.

The prospective positivity was positively associated with prospective memory (*β* = 0.27, *p* = 0.015, *η*^2^ = 0.080) (see Figure 3). The N300 was positively associated with prospective memory at a trend level (*β* = 0.20, *p* = 0.087, *η*^2^ = 0.035). All analyses controlled for age and gender.

### 3.5 Mediation analyses

**Figure 4:**
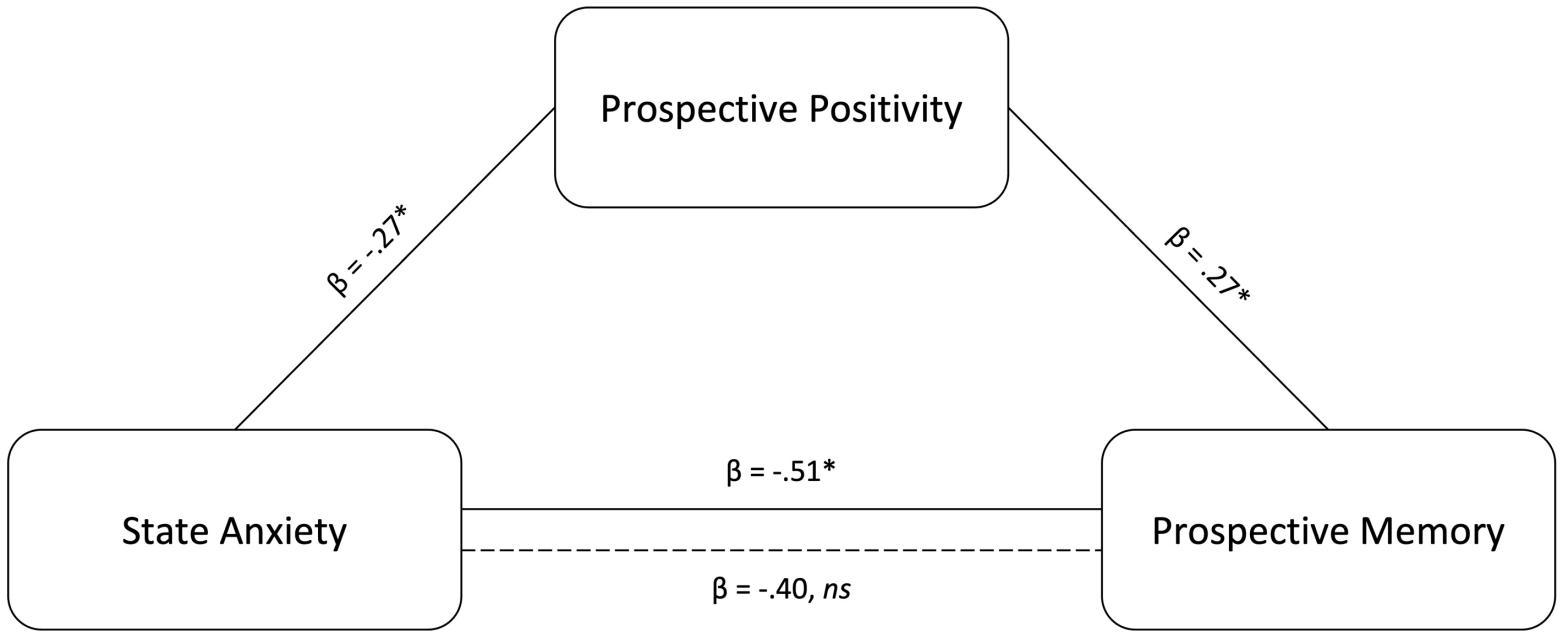
Model Results for State Anxiety Relating to Prospective Memory Accuracy via the Prospective Positivity. Mediation: State anxiety was indirectly associated with prospective memory via prospective positivity. The solid line between state anxiety and prospective memory represents the total effect (c path), while the dotted line represents the direct effect after accounting for the indirect effect (cʹ path). *p < .05.

The mediation analysis examined the prospective positivity as the mediator of the significant association between state anxiety and prospective memory while controlling for age and gender. The results showed that higher state anxiety was significantly indirectly associated with worse prospective memory (*ab* = −0.11 *p* = 0.047) via reduced prospective positivity (see Figure 4). The direct effect did not reach the *α* < 0.05 significance level (*c’* = −0.40 *p* = 0.095) but could be considered significant at a trend level. Mediation analyses were not run for the other measures of anxiety (e. g., STAI-T, BAI) because of the null main effects on prospective positivity and prospective memory.

## 4 Discussion

The current study employed EEG/ERPs to examine the relationship between PM performance, cue detection, intention retrieval, and anxiety. The findings partially support the initial hypotheses. Increased state anxiety significantly predicted decreased PM accuracy overall. State anxiety was also significantly associated with the prospective positivity. Prospective positivity then demonstrated a significant positive relationship with accuracy on PM accuracy. The N300 was significantly associated with PM accuracy at a trend level. Finally, the mediation analysis demonstrated that reduced prospective positivity partially explained the reduced PM performance in students with higher state anxiety.

### 4.1 Anxiety and prospective memory

As expected, the findings demonstrated an inverse association between anxiety and PM in this sample, replicating other studies (Arnold et al., 2015; Bowman et al., 2019). The results of the current study found that state anxiety was associated with PM, but not trait anxiety nor general anxiety. This finding suggests that PM may be more sensitive to in-the-moment, state anxiety than other forms of characteristic, long-lasting, trait anxiety. This difference could be due to the possibility that college students may struggle more to cope with immediate state anxiety, as it can be more variable and unpredictable than trait or general anxiety levels. Trait anxiety could be easier to manage due to its more stable, constant nature, which could diminish cognitive difficulties associated with this type of anxiety (Julian, 2011; Xia et al., 2020). Additional research should be carried out to fully explore potential differences in state, trait, and general anxiety for PM and other forms of cognition.

### 4.2 Anxiety and ERPs and prospective memory

The current study demonstrated increased state anxiety was associated with a blunted prospective positivity response, suggesting that college students with high state anxiety may experience disrupted activity in the neural processes that support prospective memory. While previous behavioral studies have implicated cue detection as a key component in understanding anxiety-related PM impairments (Arnold et al., 2015), the association between anxiety and N300 did not reach significance. The current study results suggest that the neural correlates of intention retrieval, usually characterized by an increased positive positivity response, may be particularly impacted by state anxiety.

Disrupted prospective positivity was further associated with decreased accuracy in the PM task, similarly to prior research that has found associations between the prospective positivity and accuracy (West, 2007), illustrating that the prospective positivity is a particularly important neural component of PM. Interestingly, the N300 was only significant with accuracy at a trend level, and the relationship was not in the expected direction: a reduced N300 response suggested higher accuracy in the PM task. While this finding is inconsistent with previous research demonstrating the importance of the N300 and cue detection in PM (West, 2007), it supports theories of spontaneous retrieval for PM goals (Einstein and McDaniel, 2005; Mullet et al., 2013). Based on the current results, it is possible that accurate performance of a PM task could occur without a strong N300 response, as an enhanced prospective positivity response can still occur.

The mediation analysis demonstrated that a reduced prospective positivity response partially explained overall reduced PM performance in students with high state anxiety. These results suggest that the neural basis of intention retrieval could play a connecting role between state anxiety and decreased PM accuracy in college students. Intention retrieval could be a key component in understanding how state anxiety interacts with PM.

Altogether, this study demonstrated that undergraduate students with high levels of state anxiety may be able to recognize when a future-intentioned task should be performed with relative ease (i.e., cue detection), but they could experience difficulty in remembering what the task was (i.e., intention retrieval). In real-world scenarios this pattern could have important implications for an anxious student’s success and well-being. For example, a college student may set a goal to study for their exam once they have returned home from their classes; while they may be able to easily recognize that they are home and they need to perform various tasks, during a period of heightened state anxiety they might have more difficulty remembering that they need to allocate time to study for their specific exam, or they may struggle to appropriately carry out the steps required to study for the exam. This impairment could result in poor academic performance and additional stressors to a student’s daily life, ultimately decreasing their success, quality of life, and well-being.

This study contributes valuable insights into the nuanced interplay between anxiety, ERPs, and PM, shedding light on the potential challenges that college students with anxiety may encounter in daily tasks requiring future-oriented memory. Understanding these cognitive dynamics is important for enhancing resources and interventions to improve the well-being and academic success of high-anxiety students navigating the demands of higher education.

### 4.3 Limitations

Results of this study should be interpreted in light of certain limitations. First, the anxiety measures in this study were based on self-reporting, resulting in subjective measurements of anxiety. While each measure has been validated by previous research, it is possible that the self-report methodology could have introduced bias into the results. The field could benefit from future studies including non-self-report measures such as clinical mental health diagnoses, heart rate variability (Jung et al., 2019), or salivary cortisol (Vreeburg et al., 2010) to limit self-report bias. Cortisol could be particularly interesting to examine, as previous work has suggested different effects of cortisol levels on PM performance, and a connection between cortisol and state anxiety (Ballhausen et al., 2019; Glienke and Piefke, 2017; Raymond et al., 2023; Schnitzspahn et al., 2022). This study was also cross-sectional, suggesting that causal conclusions cannot be assumed about the relationship between anxiety and PM from the results of this study.

Second, this study lacks racial, ethnic, and gender diversity. Both race and gender are constructs that can result in individuals encountering unique life experiences, including discrimination, social segregation, and different access to resources (Roberts et al., 2020). These different life experiences can contribute to race and gender playing a role in various psychological phenomena, including memory, emotions, executive functioning, neural activity, and psychological health (Roberts et al., 2020; Short et al., 2013; Cavanaugh and Abu Hussein, 2020; McLean et al., 2011). Relatedly, this study did not collect data on socioeconomic status, which is another factor that can play a role in differences in anxiety, brain development, and ERPs (Moriguchi and Shinohara, 2019; Perera-W.A. et al., 2021; Shahbazi et al., 2022). Results of the present study should not necessarily be assumed to be applicable to individuals of all socioeconomic backgrounds, as well as racial and gender identities. Future studies should have a more equal distribution of participants from diverse racial, ethnic, financial, and gender backgrounds.

Finally, the event-based, focal PM task used in this study is limited in its ecological validity, and it is not representative of time-based PM abilities. Since the task was focal, it may have required less monitoring for the PM cue in comparison to a non-focal task, which could have implications for assessing cue detection abilities (Cona et al., 2014; Zuber and Kliegel, 2020). While a computerized PM task was necessary to measure the desired ERP components, the nature of the task may not translate well to PM goals that college students perform in their daily lives.

### 4.4 Future directions and conclusion

While future research should improve upon the limitations, the implications of the study provide opportunities for further investigation. Intention retrieval, and overall PM performance, could be enhanced via memory support strategies or state anxiety reduction strategies, which should be investigated in future research. As state anxiety increased, the prospective positivity decreased, indicating that individuals with high state anxiety were likely experiencing difficulties with intention retrieval. These intention retrieval struggles partially explained PM accuracy detriments associated with high state anxiety. Based on these findings, future studies should investigate practical ways that intention retrieval can be strengthened in individuals with high state anxiety, such as encoding, rehearsal, and recall strategies to ease retrieving the intended goal. Further, future studies could explore how reminders can be implemented to support intention retrieval and overall PM performance. For example, how do individuals with high state anxiety utilize reminders for their future intended goals, and are some types of reminders better than other types for enhancing intention retrieval and PM? Alternatively, future studies could attempt to reduce state anxiety through various coping and self-regulation strategies to mitigate challenges with intention retrieval. In conclusion, intention retrieval could be an effective target for intervention strategies aimed at reducing the potential cognitive struggles associated with high state anxiety.

## Data Availability

The raw data supporting the conclusions of this article will be made available by the authors, without undue reservation.

## References

[ref1] American College Health Association. (2018). American College Health Association-National College Health Assessment II: Undergraduate Student Reference Group Data Report Fall 2018. Silver Spring, MD: American College Health Association.

[ref2] American College Health Association (2022). American College Health Association-National College Health Assessment III: Reference Group Executive Summary Spring 2022. Silver Spring, MD: American College Health Association.

[ref3] Andrews E. Hicks R. (2017). Dealing with anxiety: relationships among interpersonal attachment style, psychological wellbeing and trait anxiety. Int. J. Psychol. Stud. 9:53. doi: 10.5539/ijps.v9n4p53

[ref4] Aquin J. P. El-Gabalawy R. Sala T. Sareen J. (2017). Anxiety disorders and general medical conditions: current research and future directions. Focus 15, 173–181. doi: 10.1176/appi.focus.20160044, PMID: 31975849 PMC6526963

[ref5] Arnold N. R. Bayen U. J. Böhm M. F. (2015). Is prospective memory related to depression and anxiety? A hierarchical MPT modelling approach. Memory 23, 1215–1228. doi: 10.1080/09658211.2014.969276 25337864

[ref6] Ballhausen N. Kliegel M. Rimmele U. (2019). Stress and prospective memory: what is the role of cortisol? Neurobiol. Learn. Mem. 161, 169–174. doi: 10.1016/j.nlm.2019.04.010 31022446

[ref9001] Beck A. T. Epstein N. Brown G. Steer R. A. (1988). An inventory for measuring clinical anxiety: psychometric properties. Journal of consulting and clinical psychology 56:893–897. doi: 10.1037//0022-006x.56.6.893, PMID: 3204199

[ref7] Beck A. T. Steer R. A. (1993). Beck anxiety inventory manual. San Antonio, TX: Psychological Corporation.

[ref8] Bowman M. A. Cunningham T. J. Levin-Aspenson H. F. O’Rear A. E. Pauszek J. R. Ellickson-Larew S.. (2019). Anxious, but not depressive, symptoms are associated with poorer prospective memory performance in healthy college students: preliminary evidence using the tripartite model of anxiety and depression. J. Clin. Exp. Neuropsychol. 41, 694–703. doi: 10.1080/13803395.2019.1611741 31084349

[ref9] Bowman C. Cutmore T. Shum D. (2015). The development of prospective memory across adolescence: an event-related potential analysis. Front. Hum. Neurosci. 9:362. doi: 10.3389/fnhum.2015.00362, PMID: 26157379 PMC4475796

[ref10] Brandimonte M. A. Einstein G. O. Mcdaniel M. A. (2014). Prospective memory: theory and applications. New York, NY: Psychology Press.

[ref11] Cavanaugh C. Abu Hussein Y. (2020). Do journals instruct authors to address sex and gender in psychological science? Res. Integr. Peer Rev. 5:14. doi: 10.1186/s41073-020-00100-4, PMID: 33110629 PMC7583220

[ref12] Chen G. Zhang L. Ding W. Zhou R. Xu P. Lu S.. (2015). Event-related brain potential correlates of prospective memory in symptomatically remitted male patients with schizophrenia. Front. Behav. Neurosci. 9:262. doi: 10.3389/fnbeh.2015.00262, PMID: 26483650 PMC4588002

[ref13] Cona G. Arcara G. Tarantino V. Bisiacchi P. S. (2012). Electrophysiological correlates of strategic monitoring in event-based and time-based prospective memory. PLoS One 7:e31659. doi: 10.1371/journal.pone.0031659, PMID: 22363699 PMC3283681

[ref14] Cona G. Bisiacchi P. S. Moscovitch M. (2014). The effects of focal and non-focal cues on the neural correlates of prospective memory: insights from ERPs. Cerebral cortex 24, 2630–2646. doi: 10.1093/cercor/bht116 23645716

[ref9002] Cumming G. (2014). The new statistics: why and how. Psychological science 25, 7–29. doi: 10.1177/0956797613504966 24220629

[ref15] Cuttler C. Graf P. (2009). Sub-clinical compulsive checkers show impaired performance on habitual, event- and time-cued episodic prospective memory tasks. J. Anxiety Disord. 23, 813–823. doi: 10.1016/j.janxdis.2009.03.006 19394193

[ref16] Davies P. L. Segalowitz S. J. Gavin W. J. (2004). Development of response-monitoring ERPs in 7- to 25-year-olds. Dev. Neuropsychol. 25, 355–376. doi: 10.1207/s15326942dn2503_6, PMID: 15148003

[ref17] DuBois L. Z. Shattuck-Heidorn H. (2021). Challenging the binary: gender/sex and the bio-logics of normalcy. Am. J. Hum. Biol. 33:e23623. doi: 10.1002/ajhb.23623 34096131

[ref18] Einstein G. O. McDaniel M. A. (1990). Normal aging and prospective memory. J. Exp. Psychol. 16, 717–726, PMID: 2142956 10.1037//0278-7393.16.4.717

[ref19] Einstein G. O. McDaniel M. A. (2005). Prospective memory: multiple retrieval processes. Curr. Dir. Psychol. Sci. 14, 286–290. doi: 10.1111/j.0963-7214.2005.00382.x

[ref20] Glienke K. Piefke M. (2017). Stress-related cortisol responsivity modulates prospective memory. J. Neuroendocrinol. 29:544. doi: 10.1111/jne.12544 29024113

[ref21] Groot K. Strien J. W. (2019). Event-related potentials in response to feedback following risk-taking in the hot version of the Columbia card task. Psychophysiology 56:e13390. doi: 10.1111/psyp.13390, PMID: 31069812 PMC6850144

[ref22] Guillem F. Mograss M. (2005). Gender differences in memory processing: evidence from event-related potentials to faces. Brain Cogn. 57, 84–92. doi: 10.1016/j.bandc.2004.08.026, PMID: 15629219

[ref23] Guynn M. J. Mcdaniel M. A. Einstein G. O. (1998). Prospective memory: when reminders fail. Mem. Cogn. 26, 287–298. doi: 10.3758/BF03201140, PMID: 9584436

[ref24] Harris L. M. (1999). Mood and prospective memory. Memory 7, 117–127. doi: 10.1080/741943717 10645375

[ref25] Harris L. M. Cumming S. R. (2003). An examination of the relationship between anxiety and performance on prospective and retrospective memory tasks. Aust. J. Psychol. 55, 51–55. doi: 10.1080/00049530412331312874

[ref26] Hering A. Wild-Wall N. Gajewski P. D. Falkenstein M. Kliegel M. Zinke K. (2016). The role of cue detection for prospective memory development across the lifespan. Neuropsychologia 93, 289–300. doi: 10.1016/j.neuropsychologia.2016.11.008, PMID: 27847304

[ref27] Julian L. J. (2011). Measures of anxiety: state-trait anxiety inventory (STAI), Beck anxiety inventory (BAI), and hospital anxiety and depression scale-anxiety (HADS-A). Arthritis Care Res. 63, S467–S472. doi: 10.1002/acr.20561, PMID: 22588767 PMC3879951

[ref28] Jung W. Jang K. I. Lee S. H. (2019). Heart and brain interaction of psychiatric illness: a review focused on heart rate variability, cognitive function, and quantitative electroencephalography. Clin. Psychopharmacol. Neurosci. 17, 459–474. doi: 10.9758/cpn.2019.17.4.459, PMID: 31671483 PMC6852682

[ref29] Kinsella G. J. Mullaly E. Rand E. Ong B. Burton C. Price S.. (2009). Early intervention for mild cognitive impairment: a randomised controlled trial. J. Neurol. Neurosurg. Psychiatry 80, 730–736. doi: 10.1136/jnnp.2008.148346 19332424

[ref30] Kliegel M. Jäger T. (2006). The influence of negative emotions on prospective memory: a review and new data (Invited Paper) Current Psychology 4, 17. doi: 10.1007/s12144-006-1002-8

[ref31] Koo Y. W. Neumann D. L. Ownsworth T. Shum D. H. K. (2021). Revisiting the age-prospective memory paradox using laboratory and ecological tasks. Front. Psychol. 12:691752. doi: 10.3389/fpsyg.2021.691752 34220653 PMC8245680

[ref32] Ladouceur C. D. Dahl R. E. Carter C. S. (2007). Development of action monitoring through adolescence into adulthood: ERP and source localization. Dev. Sci. 10, 874–891. doi: 10.1111/j.1467-7687.2007.00639.x, PMID: 17973802

[ref33] Laera G. Arcara G. Gajewski P. D. Kliegel M. Hering A. (2021). Age-related modulation of EEG time-frequency responses in prospective memory retrieval. Neuropsychologia 155:107818. doi: 10.1016/j.neuropsychologia.2021.107818, PMID: 33675856

[ref34] Lenze E. J. Wetherell J. L. (2011). A lifespan view of anxiety disorders. Dialogues Clin. Neurosci. 13, 381–399. doi: 10.31887/DCNS.2011.13.4/elenze, PMID: 22275845 PMC3263387

[ref35] Manzi A. Nessler D. Czernochowski D. Friedman D. (2011). The development of anticipatory cognitive control processes in task-switching: an ERP study in children, adolescents, and young adults. Psychophysiology 48, 1258–1275. doi: 10.1111/j.1469-8986.2011.01192.x 21371043 PMC3130085

[ref36] McDaniel M. A. Einstein G. O. (1992). Aging and prospective memory: basic findings and practical applications. Adv. Learn. Behav. Disabilit. 7, 87–105.

[ref37] McLean C. P. Asnaani A. Litz B. T. Hofmann S. G. (2011). Gender differences in anxiety disorders: prevalence, course of illness, comorbidity and burden of illness. J. Psychiatr. Res. 45, 1027–1035. doi: 10.1016/j.jpsychires.2011.03.006, PMID: 21439576 PMC3135672

[ref38] Moriguchi Y. Shinohara I. (2019). Socioeconomic disparity in prefrontal development during early childhood. Sci. Rep. 9:2585. doi: 10.1038/s41598-019-39255-6 30796284 PMC6385208

[ref39] Mullet H. G. Scullin M. K. Hess T. J. Scullin R. B. Arnold K. M. Einstein G. O. (2013). Prospective memory and aging: evidence for preserved spontaneous retrievalwith exact but not related cues. Psychol. Aging 28, 910–922. doi: 10.1037/a0034347 24364398 PMC12327648

[ref40] Nigro G. Cicogna P. (1999). Comparison between time-based and event-based prospective memory tasks. Ricerche Psicol. 3, 55–68.

[ref41] Notebaert L. Jones E. Clarke P. J. F. MacLeod C. (2019). Trait anxiety and biased prospective memory for targets associated with negative future events. Cogn. Ther. Res. 43, 550–560. doi: 10.1007/s10608-018-9986-6

[ref42] Ordemann G. J. Opper J. Davalos D. (2014). Prospective memory in schizophrenia: a review. Schizophr. Res. 155, 77–89. doi: 10.1016/j.schres.2014.03.008 24698096

[ref43] Peirce J. Gray J. R. Simpson S. Macaskill M. Höchenberger R. Sogo H.. (2019). PsychoPy2: experiments in behavior made easy. Behav. Res. Methods 51, 195–203. doi: 10.3758/s13428-018-01193-y, PMID: 30734206 PMC6420413

[ref44] Perera-W.A. H. Salehuddin K. Khairudin R. Schaefer A. (2021). The relationship between socioeconomic status and scalp event-related potentials: a systematic review. Front. Hum. Neurosci. 15:601489. doi: 10.3389/fnhum.2021.601489 33584228 PMC7873529

[ref45] Ramos L. Miller L. van den Hoven E. (2020). Prospective memory failure in dementia: Understanding and designing to support In R. Brankaert & G. Kenning (Eds.), HCI and design in the context of dementia (Switzerland: Springer International Publishing), 131–146. doi: 10.1007/978-3-030-32835-1_9

[ref46] Raymond C. Pichette F. Beaudin M. Cernik R. Marin M. F. (2023). Vulnerability to anxiety differently predicts cortisol reactivity and state anxiety during a laboratory stressor in healthy girls and boys. J. Affect. Disord. 331, 425–433. doi: 10.1016/j.jad.2023.02.154 36972852

[ref47] Reisner S. L. Katz-Wise S. L. Gordon A. R. Corliss H. L. Austin S. B. (2016). Social epidemiology of depression and anxiety by gender identity. J. Adolesc. Health 59, 203–208. doi: 10.1016/j.jadohealth.2016.04.006 27267142 PMC4958506

[ref48] Rice M. (2023). Neural correlates of prospective memory in college students with anxiety. Master’s thesis, Colorado State University. Mountain Scholar. 10.3389/fpsyg.2024.1430373 PMC11458466 39380756

[ref49] Riess M. Janoszczyk K. Niedźwieńska A. Rendell P. G. (2016). Gender differences in prospective memory in young and older adults. Roczniki Psychol. 19, 803–812. doi: 10.18290/rpsych.2016.19.4-5en

[ref50] Roberts S. O. Bareket-Shavit C. Dollins F. A. Goldie P. D. Mortenson E. (2020). Racial inequality in psychological research: trends of the past and recommendations for the future. Perspect. Psychol. Sci. 15, 1295–1309. doi: 10.1177/1745691620927709 32578504

[ref51] Robinson O. J. Vytal K. Cornwell B. R. Grillon C. (2013). The impact of anxiety upon cognition: perspectives from human threat of shock studies. Front. Hum. Neurosci. 7:203. doi: 10.3389/fnhum.2013.00203 23730279 PMC3656338

[ref52] Schnitzspahn K. M. Plessow F. Kirschbaum C. Wong Y. H. Kliegel M. (2022). Acute psychosocial stress impairs intention initiation in young but not older adults. Psychoneuroendocrinology 135:105593. doi: 10.1016/j.psyneuen.2021.105593 34823141

[ref53] Shah T. Pol T. (2020). Prevalence of depression and anxiety in college students. J. Ment. Health Hum. Behav. 25, 10–13. doi: 10.4103/jmhhb.jmhhb_16_20

[ref54] Shahbazi F. Shahbazi M. Poorolajal J. (2022). Association between socioeconomic inequality and the global prevalence of anxiety and depressive disorders: an ecological study. Gen. Psychiatr. 35:e100735. doi: 10.1136/gpsych-2021-100735 35677849 PMC9114840

[ref55] Short S. E. Yang Y. C. Jenkins T. M. (2013). Sex, gender, genetics, and health. Am. J. Public Health 103, S93–S101. doi: 10.2105/AJPH.2013.301229, PMID: 23927517 PMC3786754

[ref56] Smith R. E. Bayen U. J. (2004). A multinomial model of event-based prospective memory. J. Exp. Psychol. Learn. Mem. Cogn. 30, 756–777. doi: 10.1037/0278-7393.30.4.756, PMID: 15238021

[ref57] Spielberger C. D. Gorsuch R. L. Lushene R. Vagg P. R. Jacobs G. A. (1983). Manual for the state-trait anxiety inventory. Palo Alto, CA: Consulting Psychologists Press.

[ref58] Tingley D. Yamamoto T. Hirose K. Keele L. Imai K. (2014). Mediation: R package for causal mediation analysis. J. Stat. Softw. 59, 1–38. doi: 10.18637/jss.v059.i0526917999

[ref59] Vreeburg S. A. Zitman F. G. van Pelt J. Derijk R. H. Verhagen J. C. van Dyck R.. (2010). Salivary cortisol levels in persons with and without different anxiety disorders. Psychosom. Med. 72, 340–347. doi: 10.1097/PSY.0b013e3181d2f0c8 20190128

[ref60] West R. (2007). The influence of strategic monitoring on the neural correlates of prospective memory. Mem. Cogn. 35, 1034–1046. doi: 10.3758/BF03193476 17910187

[ref61] West R. Herndon R. W. Covell E. (2003). Neural correlates of age-related declines in the formation and realization of delayed intentions. Psychol. Aging 18, 461–473. doi: 10.1037/0882-7974.18.3.461, PMID: 14518808

[ref62] West R. Herndon R. W. Crewdson S. J. (2001). Neural activity associated with the realization of a delayed intention. Cogn. Brain Res. 12, 1–9. doi: 10.1016/S0926-6410(01)00014-3 11489603

[ref63] West R. Krompinger J. (2005). Neural correlates of prospective and retrospective memory. Neuropsychologia 43, 418–433. doi: 10.1016/j.neuropsychologia.2004.06.012 15707617

[ref64] West R. Ross-Munroe K. (2002). Neural correlates of the formation and realization of delayed intentions. Cogn. Affect. Behav. Neurosci. 2, 162–173. doi: 10.3758/CABN.2.2.162, PMID: 12455683

[ref65] West R. Wymbs N. (2004). Is detecting prospective cues the same as selecting targets? An ERP study. Cogn. Affect. Behav. Neurosci. 4, 354–363. doi: 10.3758/CABN.4.3.354, PMID: 15535171

[ref66] Xia L. Mo L. Wang J. Zhang W. Zhang D. (2020). Trait anxiety attenuates response inhibition: evidence from an ERP study using the go/NoGo task. Front. Behav. Neurosci. 14:28. doi: 10.3389/fnbeh.2020.00028, PMID: 32218724 PMC7078641

[ref67] Zhang W. De Beuckelaer A. Chen L. Zhou R. (2019). ERP evidence for inhibitory control deficits in test-anxious individuals. Front. Psychiatr., 10–645. doi: 10.3389/fpsyt.2019.00645 PMC6743369 31551835

[ref68] Zuber S. Kliegel M. (2020). Prospective memory development across the lifespan: an integrative framework. Eur. Psychol. 25, 162–173. doi: 10.1027/1016-9040/a000380

